# The NQO1 Pro187Ser polymorphism and breast cancer susceptibility: evidence from an updated meta-analysis

**DOI:** 10.1186/1746-1596-9-100

**Published:** 2014-05-29

**Authors:** Qiliu Peng, Yu Lu, Xianjun Lao, Zhiping Chen, Ruolin Li, Jingzhe Sui, Xue Qin, Shan Li

**Affiliations:** 1 Department of Clinical Laboratory, First Affiliated Hospital of Guangxi Medical University, Nanning, Guangxi 530021, China; 2 Department of Occupational Health and Environmental Health, School of Public Health, Guangxi Medical University, Nanning, Guangxi, China; 3 Department of Medicine Research, First Affiliated Hospital of Guangxi Medical University, Nanning, Guangxi, China

**Keywords:** NQO1, Polymorphism, Breast cancer, Meta-analysis

## Abstract

**Background:**

NAD(P)H: quinone oxidoreductase 1 (NQO1) plays a central role in catalyzing the two-electron reduction of quinoid compounds into hydroquinones. The NQO1 Pro187Ser polymorphism was found to correlate with a lower enzymatic activity, which may result in increased incidence of carcinomas including breast cancer. Previous studies investigating the association between NQO1 Pro187Ser polymorphism and breast cancer risk showed inconsistent results. We performed a meta-analysis to summarize the possible association.

**Methods:**

All studies published from January 1966 to February 2014 on the association between NQO1 Pro187Ser polymorphism and breast cancer risk were identified by searching electronic databases PubMed, EMBASE, Cochrane library, and Chinese Biomedical Literature database (CBM). The association between NQO1 Pro187Ser polymorphism and breast cancer risk was assessed by odds ratios (ORs) together with their 95% confidence intervals (CIs).

**Results:**

Ten studies with 2,773 cases and 4,076 controls were finally included in the meta-analysis. We did not observe a significant association between NQO1 Pro187Ser polymorphism and breast cancer risk when all studies were pooled into the meta-analysis. In subgroup analysis by ethnicity, significant increased breast cancer risk was found in Caucasians (Ser/Pro vs. Pro/Pro: OR = 1.145, 95% CI = 1.008–1.301, P = 0.038; Ser/Ser + Ser/Pro vs. Pro/Pro: OR = 1.177, 95% CI = 1.041–1.331, P = 0.009). When stratified by source of control, significant increased breast cancer risk was found in population-based studies (Ser/Pro vs. Pro/Pro: OR = 1.180, 95% CI = 1.035–1.344, P = 0.013; Ser/Ser + Ser/Pro vs. Pro/Pro: OR = 1.191, 95% CI = 1.050–1.350, P = 0.007). However, in subgroup analyses according to menopausal status, quality score, and HWE in controls, no any significant association was detected.

**Conclusions:**

Our meta-analysis provides the evidence that the NQO1 Pro187Ser polymorphism contributed to the breast cancer susceptibility among Caucasians. Further large and well-designed studies are needed to confirm this association.

**Virtual slides:**

The virtual slide(s) for this article can be found here: http://www.diagnosticpathology.diagnomx.eu/vs/1248639991252504

## Background

Breast cancer is the most common cancer and the second most common cause of cancer-related death in women. In 2008 there were 182,460 women diagnosed with breast cancer, and 40,480 women died of this disease [1]. In several developing countries, such as China, breast cancer has surpassed cervical cancer and become the leading cause of cancer death among females [2]. Though the exact mechanism of breast carcinogenesis is still unclear, it has been well accepted that oxidative stress resulting from excess reactive oxygen species and deficiency in antioxidant capabilities play important roles in breast cancer etiology [3,4].

NAD(P)H:quinone oxidoreductase 1 (NQO1), also known as diphtheria toxin diaphorase (DT-diaphorase), is a cytosolic flavoenzyme which is present in human epithelial and endothelial tissues. NQO1 is considered as an anticancer enzyme because it protect cells from oxidative damage by preventing quinones from entering the one-electron reduction which is catalyzed by cytochrom b5 reductase or P450 reductase to generate semiquinone free radicals and reactive oxygen species [5]. On the contrary, with its unique property of transferring two electrons at a time by using either NADH or NADPH as reducing cofactor, NQO1 catalyze quinones and quinine-imines into hydroquinones, which are thought less toxic and easier to excrete when conjugated [6,7]. The NQO1 gene, mapped to chromosome 16q22.1, is 17.2 kb in length and contains 6 exons and 5 introns [8]. There were at least 270 SNPs in the NQO1 gene according to the dbSNP database (http://www.ncbi.nlm.nih.gov/SNP), including the most commonly occurring C-to-T transition at nucleotide position 609 in exon 6 (rs1800566, 609C > T), which results in a proline-to-serine amino-acid substitution at codon 187 (Pro187Ser) in the protein. It was reported that the variant T allele was associated with reduced NQO1 enzymatic activity in both human cell lines and primary human tissues [9,10]. Furthermore, there is a clear allele dosage effect of the NQO1 609 T genotypes on NQO1 enzymatic activity, with the variant homozygotes (TT) having the lowest, the heterozygotes (CT) having the intermediate, and the wild-type homozygotes (CC) having the highest NQO1 enzyme activity [11-13]. Given that the previous studies have consistently shown that the variant T allele resulted in reduced enzymatic activity, it was biologically reasonable to hypothesize a potential relationship between the NQO1 Pro187Ser polymorphism and cancer susceptibility.

In the past two decades, a number of molecular epidemiological studies have evaluated the association between the NQO1 Pro187Ser polymorphism and breast cancer risk, but the results remain inconsistent. Several studies have previously suggested that the NQO1 Pro187Ser polymorphism was associated with an increased risk of breast cancer [14,15]. However, other studies have failed to confirm such an association [3,16]. In addition, a meta-analysis by Yuan et al. [17] found that the NQO1 Pro187Ser polymorphism may contribute to breast cancer development in Caucasians. However, evidence was limited because only 6 studies were available at that time. In addition, only ethnicity was considered in the subgroup analysis and the source of heterogeneity was not explored in this study. As some new studies emerging [16,18,19], to provide the most comprehensive assessment of the associations between the NQO1 Pro187Ser polymorphism and breast cancer risk, we performed an updated meta-analysis of all available studies with extensive exploration of the source of heterogeneity and subgroup analyses.

## Methods

### Search strategy

We conducted a comprehensive literature search in PubMed, EMBASE, Cochrane library, and Chinese Biomedical Literature (CBM) databases form January 1966 to February 2014 using the following search strategy: (“breast cancer”) and (“NAD(P)H:quinone oxidoreductase 1”, or “NQO1”). There was no restriction on sample size, population, language, or type of report. All eligible studies were retrieved and their references were checked for other relevant studies. The literature retrieval was performed in duplication by two independent reviewers (Qiliu Peng and Yu Lu). When multiple publications reported on the same or overlapping data, we chose the most recent or largest population. When a study reported the results on different subpopulations, we treated it as separate studies in the meta-analysis.

### Inclusion and exclusion criteria

Studies included in the meta-analysis were required to meet the following criteria: (1) case–control or cohort studies which evaluated the association between NQO1 Pro187Ser polymorphism and breast cancer risk; (2) used an unrelated case–control design; (3) had an odds ratio (OR) with 95% confidence interval (CI) or other available data for estimating OR (95% CI); and (4) the control population did not contain malignant tumor patients. Studies were excluded if one of the following existed: (1) no control population; (2) duplicate of previous publication; and (3) insufficient information for data extraction.

### Data extraction

Two authors (Qiliu Peng and Xue Qin) independently reviewed and extracted data from all eligible studies. Data extracted from eligible studies included the first author, year of publication, ethnicity, country of origin, genotyping method, source of control, matching criteria, breast cancer ascertainment, total numbers of cases and controls and genotype frequencies of cases and controls. Ethnic backgrounds were categorized as Caucasian, Asian, and Arab. When a study did not state the ethnic descendent or if it was impossible to separate participants according to such phenotype, the group reported was termed as “mixed ethnicity”. To ensure the accuracy of the information extracted, the two authors checked the data extraction results and reached consensus on all of the items. If different results generated, they would check the data again and have a discussion to come to an agreement. If these two authors could not reach a consensus, another author (Shan Li) was consulted to resolve the dispute and a final decision was made by the majority of the votes. Menopausal status was divided into premenopausal and postmenopausal and was additionally recorded for stratified analysis.

### Quality score evaluation

The quality of eligible studies was evaluated independently by two authors (Qiliu Peng and Yu Lu) according to a set of predefined criteria (Table 1) based on the scale of Thakkinstian et al. [20]. The revised criteria cover the source of controls, representativeness of cases, ascertainment of breast cancer, total sample size, quality control of genotyping methods, and Hardy-Weinberg equilibrium (HWE) in the control population. Discrepancies were resolved by consensus. Scores ranged from 0 (lowest) to 10 (highest). Articles with scores equal to or higher than 7 were considered “high-quality” studies, whereas those with scores less than 7 were considered “low-quality” studies.

**Table 1:** Scale for quality assessment

**Criteria**	**Score**
Representativeness of cases	
Selected from cancer registry or multiple cancer center sites	2
Selected from oncology department or cancer institute	1
Selected without clearly defined sampling frame or with extensive inclusion/exclusion criteria	0
Source of controls	
Population or community based	2
Both population-based and hospital-based/healthy volunteers/blood donors	1.5
Hospital-based controls without breast cancer	1
Cancer-free controls without total description	0.5
Not described	0
Ascertainment of breast cancer	
Histological or pathological confirmation	2
Diagnosis of breast cancer by patient medical record	1
Not described	0
Sample size	
>1000	2
200-1000	1
<200	0
Quality control of genotyping methods	
Clearly described a different genotyping assay to confirm the data	1
Not described	0
Hardy-Weinberg equilibrium	
Hardy-Weinberg equilibrium in controls	1
Hardy-Weinberg disequilibrium in controls	0.5
No checking for Hardy-Weinberg disequilibrium	0

### Statistical analysis

The strength of the association between NQO1 Pro187Ser polymorphism and breast cancer risk was assessed by odds ratios (ORs) with 95% confidence intervals (CIs). The significance of the pooled OR was determined by a Z test and the p value less than 0.05 was considered significant. The association of NQO1 Pro187Ser polymorphism with breast cancer risk was assessed using co-dominant model (Ser/Ser vs. Pro/Pro and Ser/Pro vs. Pro/Pro), recessive model (Ser/Ser vs. Ser/Pro + Pro/Pro), and dominant model (Ser/Ser + Ser/Pro vs. Pro/Pro).

The χ^2^ based *Q* test was used to assess the heterogeneity among studies [21,22]. If the result of the *Q* test was *P*_*Q*_ < 0.1, indicating the presence of heterogeneity, a random-effects model (the DerSimonian and Laird method) was used to estimate the summary ORs [21]; otherwise, when the result of the *Q* test was *P*_*Q*_ ≥ 0.1, indicating the absence of heterogeneity, the fixed-effects model (the Mantel–Haenszel method) was used [22]. To explore the sources of heterogeneity among studies, we performed logistic metaregression and subgroup analyses. The following study characteristics were included as covariates in the metaregression analysis: genotyping methods (PCR-RFLP vs. not PCR-RFLP), ethnicity (Caucasians vs. not Caucasians), source of controls (Hospital-based vs. Population-based), quality scores (High-quality vs. Low-quality), HWE status (Yes vs. No), and breast cancer ascertainment (pathologically or histologically confirmed vs. other diagnosis criteria). Subgroup analyses were conducted by ethnicity, menopausal status, quality score, source of control, and HWE in controls. Galbraith plots analysis was performed for further exploration of the heterogeneity.

Sensitivity analysis was performed by sequential removal of individual studies. Publication bias was evaluated using a funnel plot and Egger’s regression asymmetry test. The distribution of the genotypes in the control population was tested for HWE using a goodness-of-fit χ^2^ test. All analyses were performed using Stata software, version 12.0 (Stata Corp., College Station, TX).

## Result

### Study characteristics

Base on our search criterion, 112 individual records were found, but only 11 full-text publications were preliminarily identified for further detailed evaluation. According to the exclusion criteria, 2 publications were excluded including 1 provide insufficient information for data extraction [23], and 1 was a meta-analysis [17]. Manual search of references cited in the published studies did not reveal any additional articles. As a result, a total of 9 relevant studies met the inclusion criteria for the meta-analysis [3,14-16,18,19,24-26]. Among them, one of the eligible studies contained data on two ethnic groups [15], and we treated it independently. Therefore, a total of 10 separate comparisons including 2,773 breast cancer cases and 4,076 controls were finally included in our meta-analysis. The main characteristics of the studies are presented in Table 2. Of all the eligible studies, 6 were conducted in Caucasian populations, 3 were in Asians, and 1 was in Arabs. Six studies were population–based and 4 were hospital–based studies. All studies used validated methods including PCR-RFLP, PCR-SSCP, PCR-CTPP, TaqMan assay, and 5′ exonuclease assay to genotype the NQO1 Pro187Ser polymorphism. The breast cancer cases were histologically or pathologically confirmed in 7 of the eligible studies. The genotype distributions of the controls in 2 studies were not consistent with HWE [16,25].

**Table 2:** Characteristics of studies included in this meta-analysis

**First author (Year)**	**Country**	**Ethnicity**	**Genotyping methods**	**Matching criteria**	**Sample size (case/control)**	**Source of control**	**BC ascertainment**	**Quality scores**	**HWE(*P *** **value)**
Yao 2013	China	Asian	TaqMan Assay	Age	162/190	HB	PC	7	0.540
Menzel Tyrol 2004	Austria	Caucasian	5′ exonuclease assay	Region	218/424	PB	HC	7.5	0.175
Menzel Prague 2004	Czech	Caucasian	5′ exonuclease assay	Age, region	190/231	PB	HC	7	0.650
Siegelmann-Danieli 2002	America	Caucasian	PCR-SSCP	Age, region	346/235	PB	HC	6	0.869
Singh 2010	India	Asian	PCR-RFLP	Age, smoking, region	200/200	HB	Other	5.5	0.000
Hamajima 2002	Japan	Asian	PCR-CTPP	NA	237/640	HB	HC	6	0.046
Sarmanova 2004	Czech	Caucasian	PCR-RFLP	Age, ethnicity	238/310	HB	HC	6	0.576
Aston 2005	America	Caucasian	PCR-RFLP	Age, region	564/1212	PB	Other	8.5	0.549
Hong 2007	America	Caucasian	TaqMan Assay	Age, ethnicity	496/495	PB	Other	7	0.531
Lajin 2013	Syria	Arab	ARMS-PCR	Ethnicity	122/139	PB	HC	7.5	0.253

BC, Breast cancer; HC, Histologically confirmed; PC, Pathologically confirmed; NA, Not available; PB, Population–based; HB, Hospital–based; HWE, Hardy–Weinberg equilibrium in control population; PCR–RFLP, Polymerase chain reaction-restriction fragment length polymorphism; PCR-SSCP, Polymerase chain reaction-single strand conformation polymorphism; PCR-CTPP, polymerase chain reaction with confronting two-pair primers; ARMS-PCR, amplification refractory mutation system-PCR.

### Meta-analysis

**Figure 1:**
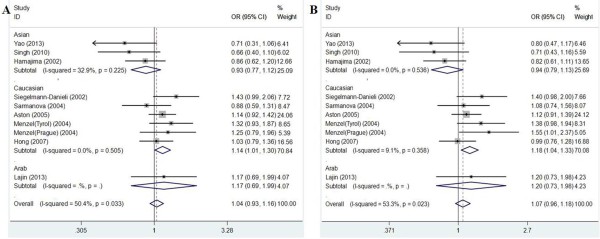
**Forest plots of the NQO1 Pro187Ser polymorphism and breast cancer risk in the overall populations. A**. Forest plot for additive model Ser/Pro vs. Pro/Pro using a fixed-effect model; **B**. Forest plot for dominant model Ser/Ser + Ser/Pro vs. Pro/Pro using a fixed-effect model.

**Figure 2:**
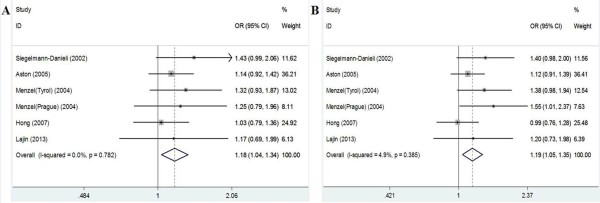
**Forest plots of the NQO1 Pro187Ser polymorphism and breast cancer risk in population-based studies. A**. Forest plot for additive model Ser/Pro vs. Pro/Pro using a fixed-effect model; **B**. Forest plot for dominant model Ser/Ser + Ser/Pro vs. Pro/Pro using a fixed-effect model.

The results of meta-analysis of the association between NQO1 Pro187Ser polymorphism and breast cancer risk were shown in Table 3. We did not observe a significant association between the NQO1 Pro187Ser polymorphism and breast cancer risk when all studies were pooled into the meta-analysis (Ser/Ser vs. Pro/Pro: OR = 1.251, 95% CI 0.843–1.856, *P* = 0.266; Ser/Pro vs. Pro/Pro: OR = 1.015, 95% CI 0.860–1.198, *P* = 0.860; Ser/Ser + Ser/Pro vs. Pro/Pro: OR = 1.058, 95% CI 0.899–1.245, *P* = 0.498; Ser/Ser vs. Ser/Pro + Pro/Pro: OR = 1.317, 95% CI 0.919–1.887, *P* = 0.133). In subgroup analysis by ethnicity, statistical significant increased breast cancer risk was found in Caucasians (Ser/Pro vs. Pro/Pro: OR = 1.145, 95% CI = 1.008–1.301, P = 0.038; Ser/Ser + Ser/Pro vs. Pro/Pro: OR = 1.177, 95% CI = 1.041–1.331, P = 0.009; Figure 1), but not in Asians and Arabs. In stratified analysis by source of control, significant increased breast cancer risk was also found in population-based studies (Ser/Pro vs. Pro/Pro: OR = 1.180, 95% CI = 1.035–1.344, P = 0.013; Ser/Ser + Ser/Pro vs. Pro/Pro: OR = 1.191, 95% CI = 1.050–1.350, P = 0.007; Figure 2), but not in hospital-based studies. However, when stratified by menopausal status, quality score, and HWE in controls, statistical significant association was not detected in all subgroups.

**Table 3:** Meta-analysis of NQO1 Pro187Ser polymorphism and breast cancer risk

**Analysis**	**No. of studies**	**Ser/Ser vs. Pro/Pro (Homozygote)**		**Ser/Pro vs. Pro/Pro (Heterozygote)**		**Ser/Ser + Ser/Pro vs. Pro/Pro (Dominant model)**		**Ser/Ser vs. Ser/Pro + Pro/Pro (Recessive model)**	
		**OR (95% CI)**	***P/P***_***Q***_	**OR (95% CI)**	***P/P***_***Q***_	**OR (95% CI)**	***P/P***_***Q***_	**OR (95% CI)**	***P/P***_***Q***_
Overall	10	1.251 (0.843-1.856)	0.266/0.004	1.015 (0.860-1.198)	0.860/0.033	1.058 (0.899-1.245)	0.498/0.023	1.317 (0.919-1.887)	0.133/0.007
Ethnicity									
Caucasian	6	1.725 (0.884-3.368)	0.110/0.003	1.145 (1.008-1.301)	0.038/0.505	1.177 (1.041-1.331)	0.009/0.358	1.654 (0.847-3.232)	0.141/0.003
Asian	3	0.785 (0.571-1.080)	0.137/0.676	0.926 (0.771-1.124)	0.309/0.225	0.941 (0.790-1.129)	0.310/0.536	1.008 (0.765-1.329)	0.955/0.195
Arab	1	1.362 (0.525-3.533)	0.526/─	1.168 (0.687-1.986)	0.565/─	1.202 (0.731-1.976)	0.469/─	1.290 (0.506-3.286)	0.594/─
Menopausal status									
Premenopausal	2	1.124 (0.681-1.853)	0.648/0.299	1.110 (0.900-1.368)	0.330/0.431	1.104 (0.902-1.352)	0.337/0.604	1.160 (0.719-1.871)	0.544/0.147
Postmenopausal	2	0.637 (0.356-1.140)	0.129/0.833	0.950 (0.737-1.225)	0.694/0.187	0.918 (0.718-1.174)	0.495/0.113	0.762 (0.445-1.305)	0.322/0.482
Quality score									
≥7	6	1.287 (0.746-2.221)	0.364/0.112	1.062 (0.864-1.307)	0.567/0.376	1.107 (0.897-1.365)	0.343/0.147	1.315 (0.817-2.117)	0.260/0.231
<7	4	1.251 (0.627-2.496)	0.524/0.021	0.942 (0.698-1.271)	0.694/0.070	0.983 (0.738-1.309)	0.906/0.071	1.368 (0.701-2.669)	0.358/0.015
Source of control									
HB	4	1.099 (0.604-2.001)	0.757/0.017	0.966 (0.823-1.140)	0.311/0.301	0.912 (0.744-1.125)	0.179/0.252	1.344 (0.784-2.304)	0.283/0.015
PB	6	1.419 (0.802-2.511)	0.230/0.124	1.180 (1.035-1.344)	0.013/0.782	1.191 (1.050-1.350)	0.007/0.385	1.335 (0.767-2.323)	0.308/0.230
HWE in controls									
Yes	8	1.450 (0.878-2.395)	0.147/0.004	1.080 (0.906-1.287)	0.389/0.069	1.136 (0.964-1.340)	0.128/0.079	1.463 (0.931-2.299)	0.099/0.011
No	2	0.791 (0.539-1.162)	0.232/0.378	0.800 (0.609-1.050)	0.108/0.389	0.788 (0.609-1.021)	0.091/0.607	0.926 (0.656-1.308)	0.663/0.105

*P*_*Q*_ P values of Q-test for heterogeneity test. OR, odds ratio; CI, confidence intervals; HB, Hospital–based studies; PB, Population-based studies; HWE, Hardy–Weinberg equilibrium.

### Test of heterogeneity

Statistical significant heterogeneity among studies was observed when all studies were pooled into the meta-analysis (Ser/Ser vs. Pro/Pro: *P*_*Q*_ = 0.004; Ser/Pro vs. Pro/Pro: *P*_*Q*_ = 0.033; Ser/Ser + Ser/Pro vs. Pro/Pro: *P*_*Q*_ = 0.023; Ser/Ser vs. Ser/Pro + Pro/Pro: *P*_*Q*_ = 0.007; Table 3). To explore the sources of heterogeneity, we performed metaregression and subgroup analyses. Metaregression analysis of data showed that the genotyping methods, source of controls, ethnicity, quality scores, HWE status, and breast cancer ascertainment were not effect modifiers of heterogeneity. Subgroup analyses stratified by ethnicity, menopausal status, quality score, source of control, and HWE status showed that heterogeneity still existed among Caucasians, low quality studies, population-based studies, and studies consistent with HWE (Table 2). To further explore the source of heterogeneity, we performed Galbraith plots analysis to identify the outliers which might contribute to the heterogeneity. Our results showed that the study Menzel et al. [15] was the outlier in the overall populations. All *P*_*Q*_ values were greater than 0.10 after excluding the study Menzel et al. [15] in the overall populations, Caucasians, population-based studies, and studies consistent with HWE. However, the significance of the summary ORs for NQO1 Pro187Ser polymorphism in the overall population and subgroup analyses were not influenced by omitting this study [15].

### Sensitivity analysis

Sensitivity analysis was performed to assess the influence of each individual study on the pooled ORs by sequential removal of individual studies. The results suggested that no individual study significantly affected the pooled ORs. In addition, sensitivity analysis was further performed by omitting the studies by Singh et al. [16] and Hamajima et al. [25] in which the control populations were not in accordance with HWE. The significance of all ORs was not altered after excluding these two studies (data not shown), indicating that our results were robust and reliable.

### Publication bias

**Figure 3:**
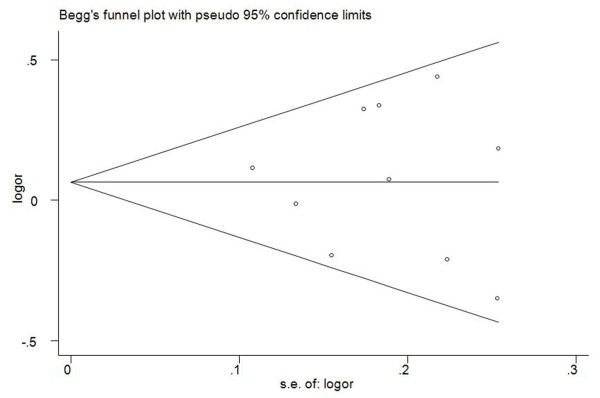
Funnel plots for publication bias of NQO1 Pro187Ser polymorphism and breast cancer risk in the overall populations (dominant model Ser/Ser + Ser/Pro vs. Pro/Pro: P = 0.704).

Begg’s funnel plot and Egger’s test were performed to assess the publication bias of literatures in all comparison models. The shape of the funnel plot did not reveal any evidence of obvious asymmetry. Then, the Egger’s test was used to provide statistical evidence of funnel plot symmetry. The results still did not suggest any evidence of publication bias (*P* = 0.114 for Ser/Ser vs. Pro/Pro; *P* = 0.277 for Ser/Pro vs. Pro/Pro; *P* = 0.704 for Ser/Ser + Ser/Pro vs. Pro/Pro, Figure 3; *P* = 0.226 for Ser/Ser vs. Ser/Pro + Pro/Pro).

## Discussion

Previous studies investigating the associations between NQO1 Pro187Ser polymorphism and breast cancer presented inconsistent results, and most of those studies involved no more than a few hundred breast cancer cases, which is too few to assess any genetic effects reliably. Meta-analysis has been recognized as an important tool to more precisely define the effect of selected genetic polymorphisms on the risk for disease and to identify potential important sources of between-study heterogeneity [27-29]. Hence, we performed this meta-analysis including all available studies to provide the most comprehensive assessment of the associations between the NQO1 Pro187Ser polymorphism and breast cancer risk. Our results showed that the NQO1 is a candidate gene for breast cancer susceptibility. The NQO1 Pro187Ser polymorphism was associated with an increased breast cancer risk among Caucasians (Ser/Pro vs. Pro/Pro: OR = 1.145, 95% CI = 1.008–1.301, P = 0.038; Ser/Ser + Ser/Pro vs. Pro/Pro: OR = 1.177, 95% CI = 1.041–1.331, P = 0.009). Our result is consistent with the previous meta-analysis performed by Yuan et al. [17].

Given the biochemical properties of NQO1 in protecting cells from oxidative damage and tumor development, this result may be biologically plausible. NQO1 is a key enzyme which catalyzes the two-electron reduction of quinoid compounds into hydroquinones, which reduces and detoxifies quinines and thus protects cells against redox cycling and oxidative stress [30,31]. Previous study suggested that some variants, especially those in the promoter regions of genes, may affect either the expression or activity levels of enzymes and therefore may be mechanistically associated with cancer risk [32]. The NQO1 Pro187Ser polymorphism is a C-to-T transition in the NQO1 gene which leads to a proline to serine amino acid substitution at codon 187 in the protein [33,34]. The NQO1 Pro187Ser polymorphism has been found correlated with decreased enzymatic activity of NQO1 and may affect host’s susceptibility to cancer by changing the enzymatic activity of NQO1 [33,34]. More importantly, the NQO1 Pro187Ser polymorphism has been shown associated with increased risk for many different types of cancers, including colorectal cancer [35], lung cancer [36], esophageal cancer [37], and hepatocellular carcinoma [38]. Our results add new evidence that the NQO1 Pro187Ser polymorphism contributes to cancer susceptibility.

In subgroup analysis stratified by ethnicity, the NQO1 Pro187Ser polymorphism presented a risk factor for breast cancer in Caucasian populations, but not in Asian and Arab subjects. The inconsistent data among the different ethnicities may indicate different effects of the NQO1 Pro187Ser polymorphism on breast risk in different ethnic genetic and environmental backgrounds. Studies reported that NQO1 enzyme not only detoxify carcinogenic compounds [6] but also bioactivate several kinds of procarcinogen [39]; thus, decreased activity of NQO1 enzyme may have dual effect on carcinogenesis. There may be some alternative ways in the Asian and Arab populations to detoxify carcinogenic compounds which can effectively compensate for the loss of NQO1 enzyme activity. Nevertheless, owing to the limited number of relevant studies among Asian and Arab populations included in the meta-analysis, the observed negative association between NQO1 Pro187Ser polymorphism and breast cancer risk in Asians and Arabs is likely to be caused by chance because study with small sample sizes may have insufficient statistical power to detect a slight effect or may have generated a fluctuated risk estimate. Currently there are only 3 studies in Asian population and 1 in Arab population for NQO1 Pro187Ser polymorphism and breast cancer risk. Therefore, the negative results of the Asain and Arab populations should be interpreted with caution.

In subgroup analysis according to the source of control, statistical significant increased breast cancer risk was found in population-based studies but not in hospital-based studies. The reason may be that the hospital-based studies have inherent selection biases due to the fact that such controls may not be representative of the study population or the general population, particularly when the genotypes under investigation were associated with the disease-related conditions that hospital-based controls may have. Thus, the use of proper and representative population-based control participants is of great importance in reducing biases in such genotype association studies.

Heterogeneity is a potential problem when interpreting the results of a meta-analysis, and finding the sources of heterogeneity is one of the most important goals of meta-analysis [40,41]. In the present study, statistical significant between-study heterogeneity among studies was observed when all studies were pooled into the meta-analysis (Ser/Ser vs. Pro/Pro: *P*_*Q*_ = 0.004; Ser/Pro vs. Pro/Pro: *P*_*Q*_ = 0.033; Ser/Ser + Ser/Pro vs. Pro/Pro: *P*_*Q*_ = 0.023; Ser/Ser vs. Ser/Pro + Pro/Pro: *P*_*Q*_ = 0.007; Table 3). To explore the sources of heterogeneity, we performed metaregression and subgroup analyses. Metaregression analysis of data showed that the genotyping methods, ethnicity, source of controls, quality scores, HWE status, and breast cancer ascertainment were not effect modifiers of heterogeneity. Subgroup analyses stratified by ethnicity, menopausal status, quality score, source of control, and HWE status showed that heterogeneity still existed among Caucasians, low quality studies, population-based studies, and studies consistent with HWE (all *P*_*Q*_ values < 0.10). Subsequently, we performed Galbraith plots analysis to further explore the source of heterogeneity. Galbraith plots analysis showed that the study Menzel et al. [15] was the outlier in all genetic models in the overall populations. When excluding the study Menzel et al. [15], the heterogeneity decreased obviously and all *P*_*Q*_ values were greater than 0.10 in all genetic comparison models in overall populations, Caucasians, population-based studies, and studies consistent with HWE. However, the summary ORs in the overall populations, Caucasians, population-based studies, and studies consistent with HWE were not material changed by omitting this study, indicating that our results were robust and reliable. The results indicated that the study Menzel et al. [15] was the major source of the heterogeneity in the meta-analysis.

However, there are still some limitations in this meta-analysis. First, in subgroup analysis by ethnicity, the included studies regarded only Caucasians, Asians, and Arabs for NQO1 Pro187Ser polymorphism. Data concerning other ethnicities such as Africans were not found. Thus, additional studies are warranted to evaluate the effect of this functional polymorphism on breast cancer risk in different ethnicities, especially in Africans. Second, our results were based on unadjusted estimates. We did not perform analysis adjusted for other covariates such as age, obesity, drinking and smoking status, use of contraceptives, environment factors, and so on, because of the unavailable original data of the eligible studies. Third, subgroup analyses were based on studies with relevant information available. Owing to the lack of detailed information in most studies, the subgroup analysis for menopausal status consisted of only two studies for each subgroup, which might not be sufficient to reach a reliable conclusion.

## Conclusions

This meta-analysis provided a more precise estimation based on larger sample size compared with the individual studies and previous meta-analysis. Our study suggested that NQO1 Pro187Ser polymorphism might contribute to breast cancer risk, especially in Caucasian populations. However, it is necessary to conduct large sample studies using standardized unbiased genotyping methods, homogeneous breast cancer patients, and well-matched controls to further validate the results of our meta-analysis. Moreover, gene-gene and gene-environment interactions should also be considered in the analysis. Such studies taking these factors into account may eventually lead to a better, more comprehensive understanding of the association between NQO1 Pro187Ser polymorphism and breast cancer risk.

## Abbreviations

HWE: Hardy–Weinberg equilibrium; NQO1: NAD(P)H: quinone oxidoreductase 1; SNP: Single nucleotide polymorphism; OR: Odds ratio; CI: Confidence interval.

## Competing interest

The authors declare that they have no competing interests.

## Authors' contributions

QP performed the literature search, data extraction, statistical analysis and drafted the manuscript. YL, XL, ZP, RL, JS, and XQ participated in data extraction. SL supervised the literature search, data extraction, statistical analysis and drafted the manuscript. All authors read and approved the final manuscript.
